# Reconstituted and frozen botulinum toxin A is as effective and safe as fresh for treating axillary hyperhidrosis: A retrospective study

**DOI:** 10.1371/journal.pone.0295393

**Published:** 2023-12-04

**Authors:** Alexander Shayesteh, Antonia Boman, Emil Hawas, Bo Carlberg

**Affiliations:** 1 Division of Dermatology and Venereology, Department of Public Health and Clinical Medicine, Umeå University, Umeå, Sweden; 2 Department of Public Health and Clinical Medicine, Umeå University, Umeå, Sweden; Fondazione Policlinico Universitario Gemelli IRCCS, ITALY

## Abstract

The use of reconstituted and frozen left-over botulinum toxin A, for treatment of patients with axillary hyperhidrosis seems to be common practice in healthcare. Thus, the objective of this study was to investigate the efficacy and safety of frozen and thawed versus fresh reconstituted abobotulinum toxin (Dysport^®^) and onabotulinum toxin (Botox^®^) in the treatment of axillary hyperhidrosis. A retrospective study was conducted analysing efficacy and data from pre- and 24 weeks post-treatment questionnaires together with medical records of individuals with moderate to severe axillary hyperhidrosis. The patients had received fresh prepared botulinum toxin A in their right axilla while frozen and thawed botulinum toxin A had been administered in their left axilla. Treatment was conducted at our Hyperhidrosis Clinic, Umeå University Hospital, Sweden 2019–2021. Pre- and post-treatment questionnaires from 106 patients were analysed. The patients were 18 to 55 years old, with a mean age of 30.7 ± 9.9 years. No significant differences in patient-reported variables, Hyperhidrosis Disease Severity Scale and VAS 10-point scale, were found between the different preparations (frozen compared to fresh) for abobotulinum toxin and onabotulinum toxin, before treatment and at 6 months follow-up. Multivariable regression analysis resulted in no significant difference regarding side-effects between the preparations or brands of botulinum toxin. The findings of this study support our clinical experience that both abobotulinum toxin and onabotulinum toxin, reconstituted, frozen and thawed, seem to be as effective and safe as fresh prepared botulinum toxin when treating axillary hyperhidrosis. Our findings indicate that left-over preparations of abo- and onabotulinum toxins, stored and frozen for up to 6 months, is a cost-and time-effective way of handling botulinum toxin for treatment of axillary hyperhidrosis.

## Introduction

Primary hyperhidrosis (PH) is a disease characterized by focal and visible excessive sweating affecting 5.5% of the population in Sweden [1]. The pathogenesis of PH is unknown however, an overstimulation of eccrine sweat glands by the sympathetic nervous system could be a potential cause of the disease [2]. While the onset of PH may be in adolescence or early childhood, a genetic predisposition has been reported in some cases [3, 4]. Common affected areas are the axillae followed by the palms, soles, and craniofacial region [5, 6]. Excessive sweating in PH has been described to have negative effects on mental and physical health and reduces quality of life [7, 8].

The diagnosis of PH can be made from the patient’s medical history [9]. Self-assessment tools, such as Hyperhidrosis Disease Severity Scale (HDSS), are used for measuring the clinical severity of the disease. HDSS is a validated international tool for assessment of hyperhidrosis [10]. HDSS is composed of one question with four answer alternatives. Each answer corresponds to a specific point yielding a total of 1–4 points. In HDSS, 1–2 points indicate mild or moderate while 3–4 points indicate severe hyperhidrosis [10]. HDSS has been translated from English to Swedish and back to English, while conceptual, item, semantic and operational equivalence of the Swedish version was discussed among researchers and patients until consensus was reached [11]. The validated Swedish HDSS was tested in PH-patients, showing a test-retest reliability with 2 weeks apart; >0.9, and an inter-rater reliability of 0.88 [12]. Objective methods for measuring sweat production, such as gravimetry, are time consuming and seldom correlate with patient assessed severity of hyperhidrosis.

Intradermal injections with botulinum toxin A (btx-A) is an effective treatment for reducing the sweat production in axillary hyperhidrosis (AH) [13, 14]. Side effects of btx-A in AH are temporary, and often described as injection related pain, compensatory sweating, muscle weakness, local infections in the skin, and flu-like symptoms [13, 14]. Treatment with btx-A must be repeated after several months, as the anhidrotic effect diminishes over time. There are two commercially available btx-A; Dysport^®^ (abobotulinum toxin (ABO), Ipsen, Inc) and Botox^®^ (onabotulinum toxin (ONA), Allergan, Inc.) which are FDA approved for treatment of AH. According to the manufacturers´ recommendations, Botox^®^ should be stored in a refrigerator (2°C– 8°C), or store in a freezer (-5°C to -20°C). After the solution is made up, immediate use of the solution is recommended; however, it can be stored for up to 24 hours in a refrigerator (2°C– 8°C). While Dysport^®^ once reconstituted, should be store in original container in a refrigerator at 2°C to 8°C and used within 24 hours. Freezing after reconstitution is not recommended [15, 16].

Prior to this investigation, other University Hospitals in Sweden, providing treatment for hyperhidrosis with ABO and ONA, were consulted regarding their management of left-over btx-A. While some clinics discarded the excess ABO and ONA within hours after preparation, the majority of clinics reported freezing the left-over btx-A syringes after reconstitution, for 1–6 months or even indefinitely. The reason provided was that no clinical loss nor any safety issues had been noticed with this practice. Thus, we decided to retrospectively investigate efficacy and safety of reconstituted, frozen and thawed compared to fresh prepared ABO and ONA which were used for treatment of axillary hyperhidrosis at our Hyperhidrosis Clinic.

## Materials and methods

### Design

A retrospective analysis of AH patients’ questionnaires and medical records from a Hyperhidrosis Clinic in Sweden.

### Setting

The Hyperhidrosis Clinic (HC) at Umeå University Hospital is an integrated part of the subsidized healthcare in Sweden and a centre for patients with hyperhidrosis in the county of Västerbotten and other counties in northern Sweden. Dermatologists at HC can see patients with hyperhidrosis via referrals. The diagnosis of hyperhidrosis is always verified before treatment. Patients are eligible for treatment if they have HDSS score of ≥ 3 points and have previously tried topical treatment without satisfactory effect. All patients with moderate to severe AH are offered treatment with btx-A injections of ABO or ONA, if no contraindications exist. Before treatment, a silicon plate with injection grids located 1.5 cm apart is used to mark the injection sites on the axillary skin, resulting in 20–25 injection sites in each axilla. Left-over and frozen btx-A was thawed only once, always administered in the left axilla, and never mixed with fresh preparations of btx-A. Since there is no data regarding safety issues for treatment with frozen and thawed btx-A in hyperhidrosis, this clinical routine aims to simplify evaluation of patients with deficient effect or reported complications from the injections. All injections were done with a 50 U (Braun, Melsungen, Germany), 0.5 ml (IU) gauge 0.3 mm x 12 mm syringes filled with 0.2–0.3 ml solution of diluted btx-A. The patients were never informed about the different preparations of btx-A although, the same commercially available btx-A is always provided in both armpits of an individual. Treatment related information such as drug labels, preparations and units given in each patient’s axilla, were always documented in the medical journals.

### Freezing procedure for btx-A

Preparing fresh syringes with btx-A for treatment of hyperhidrosis is a time-consuming procedure. The freezing duration of btx-A syringes have both practical and economic reasons. It is therefore common to prepare btx-A syringes for several consecutive patients at the same time. Patients not showing up for a visit or clinicians abstaining from treatment of some patients, result in left-over prepared btx-A syringes. Although clinicians’ approach regarding freezing and preparing botulinum toxin may vary, in this study the unused btx-A syringes were stored in a freezer within an hour, at latest after preparation. The freezing duration lasted 2 or 6 months. The btx-A syringes were stored inside letter envelopes with written text stating the name of the drug, preparation date and the number of syringes inside, being stored. This procedure aimed at preventing mistakes and helped clinicians to choose appropriate envelopes, depending on the quantity of botulinum toxin needed for a treatment session. The freezer used was an Elektro Helios, model FG 3510 freezer at constant -17° C and checked at regular intervals by an engineer.

### Data collection

Between October 2022 and November 2022, a physician reviewed and compiled data from the questionnaires and medical records of the patients collected between 2019–2021 at HC, Umeå University hospital, Sweden. The purpose of the questionnaires was to evaluate btx-A injections at HC being part of other research projects, but also to reduce unnecessary physical contacts between healthcare and patients as COVID-19 pandemic was ongoing. Hence, no objective methods of measurements were performed as it would require repeated and prolonged visits for a relatively healthy group of patients in the hospital. The coded pre-treatment questionnaire (S1 File) investigated variables such as background data, information regarding heredity, current age, age of onset for the disease, duration of effect in prior btx-A treatment for AH, previous illnesses and severity of hyperhidrosis in each axilla, assessed by HDSS and VAS 10-point scale. Patients treated with btx-A were followed-up via telephone by a nurse, using a coded 24-week post-treatment questionnaire (S2 File) to register HDSS, VAS 10-point scale, complications and potential side-effects after treatment. Data regarding the btx-A treatment (label and dose) and the corresponding questionnaires code, were obtained from the patients´ medical records.

### Statistical analysis

HC at Umeå University Hospital assesses 150–200 patients with hyperhidrosis annually. As our investigation was conducted for the years 2019–2021, we anticipated to include > 100 individuals with AH, considered sufficient for our statistical analysis. Analyses and statistical work were performed by one author not involved in assessment or treatment of patients with AH. No missing data existed among the analysed questionnaires and medical records. Data was normally distributed for HDSS, and VAS expressed as mean and standard deviation, while not normally distributed data as units of btx-A given, was expressed as median with interquartile range. The chi-square test was used for comparison of categorical variables. Paired t-test was used analysing mean values and effect data for each axilla respectively, and between baseline and the 24-week follow-up. T-test was also used for analysing differences in effect between the left and right axilla. Binary logistic regression was used for determining the impact of multiple independent variables (age, sex, freeze storage time, time for disease onset, co-existing diseases, units given in each axilla and HDSS outcome for frozen and thawed btx-A treated axilla) to predict reported side-effects as dependent variable. Two-sided p-values ≤ 0.05 was considered statistically significant. Calculations of statistical analyses were performed in IBM SPSS Statistics for Mac (version 28). Relevant and anonymized data that was used for statistical analyses can be found as S1 Data.

### Ethical considerations

Ethical approval for research related to human subjects was granted by the Swedish Ethical Review Authority (EPN) (Dnr. 2010-199-31M & Dnr. 2020–01253). A code, always assigned to each patient’s pre-treatment questionnaire, was used for registering data in the post-treatment questionnaire. Oral and written informed consent for the questionnaires, were obtained on the day of the visit at the hyperhidrosis clinic.

## Results

We were able to access pre- and post-treatment questionnaires of 111/121 AH patients treated with btx-A, who had given their consent for the study. Due to crucial missing data, concerning coding related issues and answers missing, 5/111 questionnaires were excluded. The study population (n = 106) consisted of patients treated with ABO n = 54 (50.9%) and ONA n = 52 (49.1%). The age of the participants ranged from 18 to 55, with a mean age of 30.7 ± 9.9 years. Revisiting patients were 91.5% of all patients. The revisiting patients had received treatment with Dysport^®^ for AH >1 year prior to the visit and were well acquainted with the effect and side-effects of this treatment. The only significant difference in background characteristics between sexes was the onset of AH being earlier in women (14.9 ± 3.7, p<0.001) (Table 1).

**Table 1:** Characteristics of the study population with axillary hyperhidrosis.

	Participants	Men	Women
N	106	27	79
Age, years, mean ± SD	30.7 ± 9.9	33.0 ± 10.4	29.9 ± 9.7
Age at onset, years, mean ± SD	15.4 ± 4.3***	17.0 ± 5.3	14.9 ± 3.7
Co-existing diseases, n (%)	32 (30.2)	7 (25.9)	25 (31.6)
Prior treatment with btx-A, n (%)	97 (91.5)	25 (92.6)	72 (91.1)
Abobotulinum toxin, n (%)	54 (50.9)	14 (51.9)	40 (50.6)
*Units per axilla*, *median (IQR)*	91.0 (15.0)	91.4 (21.0)	88.1 (15.5)
Onabotulinum toxin, n (%)	52 (49.1)	13 (48.1)	39 (49.4)
*Units per axilla*, *median (IQR)*	44.0 (4.0)	45.7 (3.4)	43.0 (3.8)

IQR, interquartile range; SD, standard deviation.

*p <0.05,

**p<0.01,

***p<0.001

The difference in effect-size measured by HDSS, was not significant between the axillae, at 24 weeks follow-up for ABO, stored either frozen for two (p = 0.33) or six months (p = 0.57). Also, no significant difference was found in effect-size between the axillae for ONA, stored frozen for 2 or 6 months (p = 1.00 respectively). The mean effect-size between the two- and six-months freeze stored btx-A, was not significant for ABO (p = 0.90) nor for ONA (p = 0.23) (Table 2).

**Table 2:** Difference in mean effect-size between fresh prepared vs. frozen and thawed abobotulinum toxin and onabotulinum toxin.

Btx-A	n	Freezer stored btx-A	Follow-ups	HDSS^a^ left and right axilla
ABO	26	2 months	24 weeks	1.25±1.11 vs 1.29±1.16
ONA	27			1.25±0.93 vs 1.24±0.90
ABO	28	6 months	24 weeks	1.21±0.99 vs 1.25±1.01
ONA	25			0.91±1.07 vs 0.90±1.06

HDSS, Hyperhidrosis Disease Severity Scale; VAS, visual analogue scale.

^a^ paired t-test comparing effect-size left axilla (frozen and thawed btx-A) and right axilla (fresh btx-A)

*p <0.05,

**p<0.01,

***p<0.001

Fresh prepared, and all preparations of frozen and thawed (2 and 6 months) ABO and ONA had significant effect (p<0.001) in reducing hyperhidrosis measured by HDSS and VAS 24 weeks after treatment. In total, 15/106 (14.3%) patients reported side-effects from the treatment such as sensations of burning, pain or itching at the injection sites (n = 3), weakness in the muscles of the hands (n = 4), bromhidrosis (n = 1), dizziness (n = 2), and compensatory sweating (n = 5). All side-effects were temporary and reported to have diminished in < 1 month. There were no significant differences in reported side-effects between ABO and ONA (p = 0.87), nor between frozen and thawed versus fresh btx-A for ABO (p = 0.68) or ONA (p = 0.92). Furthermore, there was no significant difference between men and women, regarding experienced side-effects from ABO or ONA (p = 0.348 and p = 0.840, respectively) (Table 3).

**Table 3:** Summary of binary logistic regression analysis for patient-reported side-effects.

Variable	B	S.E.	OR	95 CI for OR
Age	0.02	0.03	1.02	0.56–1.09
Sex	0.33	0.73	1.39	0.33–5.79
Freezer stored time of btx-A	-0.62	0.62	0.54	0.16–1.79
Age of onset	0.05	0.08	1.05	0.89–1.23
Co-existing diseases	0.44	0.73	1.55	0.37–6.54
Units btx-A given	0.01	0.01	1.00	0.98–1.03
HDSS left axilla 24 w	0.58	0.39	1.79	0.83–3.85

Abbreviations: CI, confidence interval; SE, standard error; OR odds ratio; btx-A, botulinum toxin A; HDSS, Hyperhidrosis Disease Severity Scale; w, weeks.

*p <0.05,

**p<0.01,

***p<0.001

## Discussion

This is, to our knowledge, the only study from a dermatological clinic which provides data regarding clinical effect and safety of frozen and thawed, compared to fresh prepared ABO (Dysport^®^) and ONA (Botox^®^). Our findings stress the experiences from healthcare that both ABO and ONA are clinically effective, after reconstitution, freezing, thawing and administration in patients with AH. In addition, the administered frozen (2 & 6 months) and thawed btx-A was safe, without significant differences in reported side-effects compared with fresh prepared btx.

Discarding unused portions of reconstituted botulinum toxin solutions is impractical and results in extensive economic losses. Although freezing and thawing btx-A is not recommended by the manufacturers IPSEN and Allergan, it is being common in healthcare and there are smaller trials with results supporting this practice [17–20]. The most recent study investigating potency and quality of a btx-A; Prabotulinum toxin A, reported that the drug was stable after 48, 24, and 12 weeks, when stored at freezing, refrigeration, or room temperatures, respectively which confirms our findings [21]. Storing and administrating medications in extreme temperatures has been reported and sometimes necessary to perform [22]. The efficacy and safety of this practice could widely vary between different drugs, from more temperature-sensitive, to more stable ones. Extreme heat or sub-zero temperatures can potentially cause degradation, chemical reactions such as oxygenation, hydrolysis or decarboxylation to any medication [23, 24]. As data regarding this topic is sparse, we can only speculate that factors such as temperature, time for the toxin solution to freeze, diameter of the vial containing btx-A solution, duration of storage of frozen solution and factors related to thawing process of the btx-A solution, could play different roles in degradation of the toxins and thereby, affecting the clinical outcome of a treatment. The result of this study is contradictory to the general hypothesis that there would be a significant degradation of btx-A if frozen thereby, affecting the clinical effect experienced by patients. In a study by Gartlan et al., it was reported that ONA frozen and stored for 2 weeks, resulted in a significant reduction of its potency, when refrigerated for 12 hours but not for 6 hours [18]. The freezing and storage temperature used by Gartlan et al. was -70° Celsius, while the btx-A is frozen and stored in -17° Celsius. Also, the procedure of immediately using the prepared btx-A when thawed, could have impact on protein degradation and minimize the risk of an insufficient clinical effect.

Another consideration is that most of the participants (91.5%) had prior experience regarding clinical effect, effect-duration and side-effects of btx-A injections for their AH. While it has been reported that the placebo-effect, in dermatological treatments could be high [25], our patients should have recognized and reacted to a suboptimal clinical effect of frozen and thawed btx-A injection. As this was not reported at the 24 weeks follow-up, the placebo-effect cannot by itself explain our findings.

The low onset age of axillary hyperhidrosis among female participants was also an interesting finding. While data regarding this matter is sparse, Hamm et al. [7] reported a later onset of axillary compared to palmar hyperhidrosis, both among men and women. A hypothesis provided was that AH may be triggered by factored related to physiological or hormonal changes associated with puberty. This is a reasonable suggestion as puberty could be initiated earlier in girls, which would also explain our findings. More epidemiological data regarding hyperhidrosis is necessary.

Finally, in dermatological practice, the intradermal injections of botulinum toxins are often performed by penetrating the skin only one to a few millimeters. While post-treatment side-effects involving skin infections were not reported by our patients, one must consider that reconstituting and freezing prepared syringes of medication may cause contamination with bacteria, and potentially cause an infection if administered into the skin. In a study, investigating bacterial and fungi contamination in used vials of btx-A; Botox, while refrigerated for 4 weeks, no detectable contamination was observed [26]. Important methods for reducing contamination of syringes and needles during preparation are primarily wearing protective gloves, using experienced staff, and having protective caps on needles after preparation [27]. World Health Organization recommends that if a prepared injection cannot be administered immediately, a cap should cover the needle and the syringe is to be stored in a kidney dish or a similar container [28] which is the routine in healthcare. However, we were not able to find any studies investigating the infection risk according to the procedures described in our study. While it is our clinical experience that our method of freezing prepared syringes is common in healthcare, great care is needed to evaluate potential risks and benefits, when injecting structures in the body such as deep muscles. While there were no reported clinical skin infections in our sample, it would be prudent to assume that an infection risk still exists and potential infection symptoms from patients should be taken seriously.

### Strengths and limitations

The standardized care and routines at our HC were crucial enabling us to conduct this retrospective analysis. Also, the possibility of having side-to-side comparison of the patient’s left and right axilla added reliability to our findings since potential confounding are usually avoided this way. A limitation could have been the absence of objective quantification of sweat production before and after treatment. However, it must be considered that methods such as gravimetry are time consuming and seldom used in clinical practice and that data obtained for this study was collected during the COVID19 pandemics in which patient examinations not related to disease assessment or treatments were restricted. As HDSS and VAS are validated and international scales, often used in evaluation of patients with hyperhidrosis, the findings of this study reflect and confirms standard clinical practices in healthcare in assessment of patients with hyperhidrosis.

Finally, deficient data regarding potency and efficacy of btx-A, limit us to compare our findings. Thus, A non-inferiority study, investigating different preparations and storage procedures for btx-A, and measuring subjective and objective outcomes in patients, is warranted to further strengthen and confirm evidence of freezing and thawing prepared btx-A in hyperhidrosis.

## Conclusions

In conclusion, our findings support the general perception within healthcare, that both btx-A, ABO and ONA, seem to be safe and able to retain their clinical potency, after preparation freeze storage and thawing for administration in patients. Thus, freezing and storing botulinum toxin A for later use, instead of discarding the prepared and leftover syringes containing btx-A, could be a cost-effective way to treat AH, hence enabling more patients to benefit from this treatment. In addition, the procedure of thawing reconstituted and frozen left-over btx-A, instead of using fresh prepared toxins for treatment, spares time and simplifies handling treatment for AH within healthcare.

## Supporting information

S1 File Hyperhidrosis pre-treatment questionnaire. (PDF)

S2 File Hyperhidrosis post-treatment questionnaire. (PDF)

S1 Data (XLSX)
