# Supplementary material for: Reconstituted and frozen botulinum toxin A is as effective and safe as fresh for treating axillary hyperhidrosis: A retrospective study
Source: PLoS One. 2023 Dec 4;18(12):e0295393. doi: 10.1371/journal.pone.0295393 (PMC10695379; doi:10.1371/journal.pone.0295393)
Supplement: S1 File — (PDF) [file pone.0295393.s001.pdf]

Pre-treatment questionnaire

Code:……. Date:……………………

Age:……..years Sex: □Male □Female □Other □I don´t want to answer

1. Where on your body do you suffer from hyperhidrosis (excessive sweating)? (choose one option)

□ feet □ palms □ axilla □ face □ other____________________

2.How old were you when the hyperhidrosis started? ________ years

3.Do you have any other diseases?

□ No □ Yes ____

4.Have you previously been treated with botulinum toxin injections due to hyperhidrosis?

□ No □ Yes and the effect lasted approximately______ months

5. How would you rate the severity of hyperhidrosis, *before treatment* on your **left side**:

0 1 2 3 4 5 6 7 8 9 10

No symptoms |—|—|—|—|—|—|—|—|—|—| Intolerable

6. How would you rate the severity of hyperhidrosis, *before treatment* on your **right side**:

0 1 2 3 4 5 6 7 8 9 10

No symptoms |—|—|—|—|—|—|—|—|—|—| Intolerable

7. How would you rate the severity of your hyperhidrosis **(left side)**?
□ My sweating is never noticeable and never interferes with my daily activities
□ My sweating is tolerable but sometimes interferes with my daily activities
□ My sweating is barely tolerable and frequently interferes with my daily activities
□ My sweating is intolerable and always interferes with my daily activities

8. How would you rate the severity of your hyperhidrosis (**right side**)?
□ My sweating is never noticeable and never interferes with my daily activities
□ My sweating is tolerable but sometimes interferes with my daily activities
□ My sweating is barely tolerable and frequently interferes with my daily activities
□ My sweating is intolerable and always interferes with my daily activities

9.Would you like to add more comments?_______________________________________
____
____
