# Supplementary material for: Reconstituted and frozen botulinum toxin A is as effective and safe as fresh for treating axillary hyperhidrosis: A retrospective study
Source: PLoS One. 2023 Dec 4;18(12):e0295393. doi: 10.1371/journal.pone.0295393 (PMC10695379; doi:10.1371/journal.pone.0295393)
Supplement: S2 File — (PDF) [file pone.0295393.s002.pdf]

Post-treatment questionnaire

Code:……. Date:……………………

Age:……..years Sex: □Male □Female □Other □Non

1. Where on your body did you receive treatment with botulinum toxin?

□ feet □ palms □ axilla □ face □ other___________________

2. Botulinum toxin type given □ Dysport □ Botox

3. Botulinumtoxin units given in each axilla: ………U Left and ………U Right

4. How would you rate the severity of hyperhidrosis, *today* on your **<u>left side</u>**:

0 1 2 3 4 5 6 7 8 9 10

No symptoms |—|—|—|—|—|—|—|—|—|—| Intolerable

5. How would you rate the severity of hyperhidrosis, *today* on your **<u>right side</u>**:

0 1 2 3 4 5 6 7 8 9 10

No symptoms |—|—|—|—|—|—|—|—|—|—| Intolerable

6. How would you rate the severity of your hyperhidrosis **(left side)**?
□ My sweating is never noticeable and never interferes with my daily activities
□ My sweating is tolerable but sometimes interferes with my daily activities
□ My sweating is barely tolerable and frequently interferes with my daily activities
□ My sweating is intolerable and always interferes with my daily activities

7. How would you rate the severity of your hyperhidrosis (**right side**)?
□ My sweating is never noticeable and never interferes with my daily activities
□ My sweating is tolerable but sometimes interferes with my daily activities
□ My sweating is barely tolerable and frequently interferes with my daily activities
□ My sweating is intolerable and always interferes with my daily activities

8. Have you experienced any side-effects related to your treatment?

□ No □ Yes ____

9. 7. Have you experienced any complications related to your treatment?

□ No □ Yes ____

10. Would you like to add any more comments?

____

____
